# The Prognostic Impact of Tumor Architecture for Upper Urinary Tract Urothelial Carcinoma: A Propensity Score-Weighted Analysis

**DOI:** 10.3389/fonc.2021.613696

**Published:** 2021-02-25

**Authors:** Hui-Ying Liu, Yen Ta Chen, Shun-Chen Huang, Hung-Jen Wang, Yuan-Tso Cheng, Chih Hsiung Kang, Wei Ching Lee, Yu-Li Su, Chun-Chieh Huang, Yin-Lun Chang, Yao-Chi Chuang, Hao Lun Luo, Po Hui Chiang

**Affiliations:** ^1^ Department of Urology, Kaohsiung Chang Gung Memorial Hospital and Chang Gung University College of Medicine, Kaohsiung, Taiwan; ^2^ Department of Pathology, Kaohsiung Chang Gung Memorial Hospital and Chang Gung University College of Medicine, Kaohsiung, Taiwan; ^3^ Department of Hematology and Oncology, Kaohsiung Chang Gung Memorial Hospital and Chang Gung University College of Medicine, Kaohsiung, Taiwan; ^4^ Department of Radiation Oncology, Kaohsiung Chang Gung Memorial Hospital and Chang Gung University College of Medicine, Kaohsiung, Taiwan

**Keywords:** tumor architecture, papillary, sessile, upper urinary tract urothelial carcinoma, metastasis, cancer-specific survival

## Abstract

**Purpose:**

To assess the association of tumor architecture with cancer recurrence, metastasis, and cancer-specific survival (CSS) in patients treated with radical nephroureterectomy (RNU) for upper urinary tract urothelial carcinoma (UTUC) in Taiwan.

**Materials and Methods:**

Data were collected from 857 patients treated with RNU between January 2005 and August 2016 in our hospital. Pathologic slides were reviewed by genitourinary pathologists. Propensity score weighting was performed for data analysis.

**Results:**

Sessile growth pattern was observed in 212 patients (24.7%). Tumor architecture exhibited a significant association with bladder cancer history, chronic kidney disease (CKD), tumor stage, lymph node status, histological grade, lymphovascular invasion, concomitant carcinoma *in situ*, and the variant type [standardized mean difference (SMD) > 0.1 for all variables before weighting]. In the propensity score analysis, 424 papillary and sessile tumor architecture were analyzed to balance the baseline characteristics between the groups. Tumor architecture was an independent predictor of metastatic disease and CSS (p = 0.033 and p = 0.002, respectively). However, the associations of tumor architecture with bladder and contralateral recurrence were nonsignificant (p = 0.956 and p = 0.844, respectively).

**Conclusions:**

Tumor architecture of UTUC after RNU is associated with established features of aggressive disease and predictors of metastasis and CSS. Assessment of tumor architecture may help identify patients who could benefit from close follow-up or early administration of systemic therapy after RNU. Tumor architecture should be included in UTUC staging after further confirmation.

## Introduction

Upper urinary tract urothelial carcinoma (UTUC), referred to as renal pelvic and ureteral tumors, comprises approximately 5% of all urothelial tumors and 10% of renal tumors (1–7). The incidence and biological behaviour of UTUC vary across ethnicities and geographic areas (8). Based on the Surveillance, Epidemiology and End Results database, the incidence of UTUC in the United States has been reported as low as 2.06 cases per 100,000 person-years (7). However, the incidence and disease presentation of UTUCs in the Asian population, particularly in Taiwan, differ from those in the Western population (9–12). First, UTUC accounts for 20%–30% of urothelial tumors and is more common in Asian than in Western populations (9, 12). Second, a high prevalence of non-organ confined (43%) and high-grade (82%) disease in Asiatic patients has been reported (10, 11). Third, UTUCs are more common in female than in male patients (9, 12, 13). Finally, in Asian countries, female patients with UTUC are less likely to develop late stage, large-sized tumor, and lymph node metastasis (LNM) than male patients, whereas this difference is not observed in Western countries (9).

Radical nephroureterectomy (RNU) with bladder cuff excision is the standard treatment for UTUC (1–7). For patients at high risk of treatment failure with RNU alone, adjuvant therapies are reasonable (6, 14). Of the total number of patients with UTUC, a substantial proportion of patients experience disease recurrence and 20%–55% may develop metastases and subsequently die from the disease (3, 6). The disease stage is the most important prognostic factor for UTUC (4). Identification of the clinical stage and prognosis are essential for accurate assessment and clinical decision-making for patients (1, 6).

Tumor stage, histologic grade, and LNM are the well-established and significant prognostic factors (1–3, 9). Several studies have evaluated the possible predictive factors for cancer recurrence and survival after RNU (1, 2, 6). The oncologic significance of other potentially relevant variables, such as tumor architecture, tumor site, tumor necrosis, lymphovascular invasion (LVI), and concomitant carcinoma *in situ* (CIS) remain to be confirmed (2, 6). The role of adjuvant chemotherapy was considered a new standard of care for patients with locally advanced UTUC to improve outcome (14). The sessile tumor architecture has been reported to be a predictor of poor outcomes in patients with bladder urothelial carcinoma (UC), and several studies have also investigated the significance of tumor architecture in patients with UTUC (3, 5, 6). Recognising these limitations, we report a large series from Taiwan, an endemic area of UTUC, to assess whether tumor architecture could be a valuable parameter for refining the prognosis of patients with UTUC.

## Materials and Methods

### Study Population

Between January 2005 and December 2016, a total of 1,077 patients with localized upper urinary tract cancer were administered surgical intervention at our institution. Of the total, we excluded 178 patients who underwent nephron-sparing surgery and 42 patients with non-UC histology. Overall, we included 857 patients who underwent nephroureterectomy with bladder cuff excision at our institution to assess the prognostic significance of tumor architecture in the clinical course of localized UT-UC. All enrolled patients underwent cystoscopy or computed tomography (CT) to preoperatively observe the presence of concurrent bladder disease or distant metastasis. We performed lymph node dissection only when lymph node was larger than 1cm from pre-operative imaging or suspicious lesions during operation. The percentage of negative lymph nodes was defined as negative pathological findings after lymph node dissection or patient did not underwent lymph node dissection, which was 85.1% in the papillary tumor architecture group and 75.5% in the sessile tumor architecture group. Perioperative data, such as age, sex, smoking history, and bladder cancer history, were obtained through chart review. This study was approved by the Institutional Review Board of Kaohsiung Chang Gung Memorial Medical Center (IRB number: 202000185B0).

### Pathological Evaluation

UC was histologically confirmed in all specimens, and specimens with variant histology were also included in this study. Genitourinary pathologists, who were blinded to the clinical outcomes, reviewed all slides according to identical strict criteria. Tumors were staged according to the American Joint Committee on Cancer tumor–node–metastasis (TNM) classification. Tumor grading was assessed according to the 2004 and 2016 World Health Organisation/International Society of Urologic Pathology consensus classification (15–18). Tumor architecture was defined by a uropathologist at our institution based on the predominant feature (3, 19). Tumor stage, architecture, grade, necrosis, and concomitant CIS were also assessed in every representative slide.

### Follow-Up Protocol and Definition of Oncological Event

Our institutional follow-up protocol included postoperative cystoscopy every 3 months. CT was performed annually to assess lymph node status and local or regional recurrence of the tumor. Elective bone scans, chest CT, and magnetic resonance imaging were performed when clinically indicated. Metastasis was defined as local failure in the operative site or regional lymph nodes or distant metastasis. Bladder and contralateral recurrences were considered separately in the analysis of recurrence-free survival. Treating physicians determined the cause of death by using chart review or by inspecting death certificates. Cancer-specific death was defined as death event due to concurrent UC metastases or progressive disease.

### Statistical Analysis

Descriptive statistical analysis results of continuous variables were reported as mean and standard deviation, and data for categorical variables in the study cohort were summarized as n (%). To address systematic differences between sessile and papillary groups (i.e., the confounding baseline parameter factor), we applied the average treatment effect for the treated (ATT) units weighting analysis (also called weighting by odds).

ATT, a form of propensity-score analysis, can be used in outcome analysis to estimate the average treatment effect for the treated units (individuals who actually received the treatment) by weighting the control group to the treated group. The propensity score was calculated using logistic regression to model the tumor architecture in the baseline period by age at index date, sex, bladder cancer history, tumor location, chronic kidney disease (CKD) group, cancer stage, lymph node status, histology grade, lymphovascular invasion, CIS, tumor necrosis, variant type, and perioperative chemotherapy.

The algorithm combined weighted estimates across several parametric and nonparametric prediction modelling approaches based on the accuracy of predictions from the models to create an overall propensity score estimate, which increased the robustness of the analysis. Postweighting balance in covariates between treatment groups was evaluated using the standardised mean difference (SMD) approach. Imbalance was defined as a standardised mean difference (SMD) of >0.1.

Kaplan–Meier curves and log-rank tests were used to compare metastasis-free survival (MFS), cancer-specific survival (CSS), bladder recurrence-free survival, and contralateral recurrence-free survival between two tumor architecture groups with and without ATT weighting. All statistical tests were two-tailed and conducted at 5% significance level by using R version 3.6.3 and the IPW survival, tableone, survey, and hrIPW packages.

## Results

### Association of Tumor Architecture With Clinical and Pathologic Characteristics

**Table 1** shows the association of tumor architecture with clinical and pathologic characteristics before and after propensity score matching (PSM). Of 857 patients, sessile and papillary growth patterns were present in 212 (24.7%) and 645 (75.3%) patients, respectively. The mean follow-up period of sessile group and papillary group was 39.34 ± 35.31 months and 47.10 ± 35.19 months, respectively. Tumor architecture exhibited significant association with bladder cancer history, CKD group, tumor stage, lymph node status, histological grade, LVI, concomitant CIS, and variant type (SMD > 0.1 for all variables before weighting). In total, 32 (5.0%) patients with papillary growth pattern and 36 (17.0%) patients with sessile growth pattern had received either neoadjuvant or adjuvant perioperative chemotherapy. In the propensity score analysis, 424 papillary and sessile tumor architecture were analysed. The baseline characteristics in the weighted groups were well balanced.

**Table 1:** Association of tumor architecture with clinical and pathologic characteristics in patients treated with radical nephroureterectomy for upper tract urothelial carcinoma before and after propensity-score analysis.

Characteristic	Before Weighting			After Weighting		
	Papillary (n = 645)	Sessile (n = 212)	SMD	Papillary (n = 212)	Sessile (n = 212)	SMD
**Follow-up, month, [mean(SD)]**	47.10 (35.19)	39.34 (35.31)				
**Age, [mean (SD)]**	66.80 (10.84)	67.18 (9.81)	0.036	66.89 (10.45)	67.18 (9.81)	0.028
**Gender, n (%)**			0.038			0.040
**Men**	295 (45.7%)	101 (47.6%)		105 (49.6%)	101 (47.6%)	
**Women**	350 (54.3%)	111 (52.4%)		107 (50.4%)	111 (52.4%)	
**Bladder cancer history, n (%)**			0.134			0.026
**Negative**	468 (72.6%)	166 (78.3%)		168 (79.4%)	166 (78.3%)	
**Positive**	177 (27.4%)	46 (21.7%)		44 (20.6%)	46 (21.7%)	
**Cancer location, n (%)**			0.049			0.003
**Multifocal**	175 (27.1%)	53 (25.0%)		53 (24.9%)	53 (25.0%)	
**Unifocal**	470 (72.9%)	159 (75.0%)		159 (75.1%)	159 (75.0%)	
**CKD Group, n (%)**			0.244			0.079
**Stage 1**	53 (8.2%)	20 (9.4%)		20 (9.6%)	20 (9.4%)	
**Stage 2**	147 (22.8%)	62 (29.2%)		68 (32.3%)	62 (29.2%)	
**Stage 3**	225 (34.9%)	80 (37.7%)		73 (34.4%)	80 (37.7%)	
**Stage 4**	72 (11.2%)	17 (8.0%)		18 (8.3%)	17 (8.0%)	
**Stage 5**	148 (22.9%)	33 (15.6%)		33 (15.4%)	33 (15.6%)	
**Stage, n (%)**			0.742			0.001
**Localized**	486 (75.3%)	87 (41.0%)		87 (41.0%)	87 (41.0%)	
**Locally advanced**	159 (24.7%)	125 (59.0%)		125 (59.0%)	125 (59.0%)	
**LN status, n (%)**			0.240			0.008
**Negative**	624 (96.7%)	193 (91.0%)		192 (90.8%)	193 (91.0%)	
**Positive**	21 (3.3%)	19 (9.0%)		20 (9.2%)	19 (9.0%)	
**Histological grade, n (%)**			0.250			0.006
**Low**	65 (10.1%)	8 (3.8%)		8 (3.7%)	8 (3.8%)	
**High**	580 (89.9%)	204 (96.2%)		204 (96.3%)	204 (96.2%)	
**LVI, n (%)**			0.522			0.027
**Negative**	529 (82.0%)	125 (59.0%)		128 (60.3%)	125 (59.0%)	
**Positive**	116 (18.0%)	87 (41.0%)		84 (39.7%)	87 (41.0%)	
**Concomitant CIS, n (%)**			0.149			0.007
**Negative**	403 (62.5%)	117 (55.2%)		116 (54.8%)	117 (55.2%)	
**Positive**	242 (37.5%)	95 (44.8%)		96 (45.2%)	95 (44.8%)	
**Tumor necrosis, n (%)**			0.055			0.001
**Negative**	409 (63.4%)	140 (66.0%)		140 (66.0%)	140 (66.0%)	
**Positive**	236 (36.6%)	72 (34.0%)		72 (34.0%)	72 (34.0%)	
**Variant type, n (%)**			0.128			0.038
**Negative**	435 (67.4%)	130 (61.3%)		126 (59.5%)	130 (61.3%)	
**Positive**	210 (32.6%)	82 (38.7%)		86 (40.5%)	82 (38.7%)	
**Periop CT (ACT+NCT), n (%)**			0.392			0.029
**Negative**	613 (95.0%)	176 (83.0%)		178 (84.1%)	176 (83.0%)	
**Positive**	32 (5.0%)	36 (17.0%)		34 (15.9%)	36 (17.0%)	

SMD, standardized mean difference; SD, standard deviation; CKD, chronic kidney disease; LN, lymph node; LVI, lymphovascular invasion; CIS, carcinoma in situ; CT, chemotherapy; ACT, adjuvant chemotherapy; NCT, neoadjuvant chemotherapy.

### Association of Tumor Architecture With Clinical Outcomes

#### Bladder Recurrence

**Figure 1:**
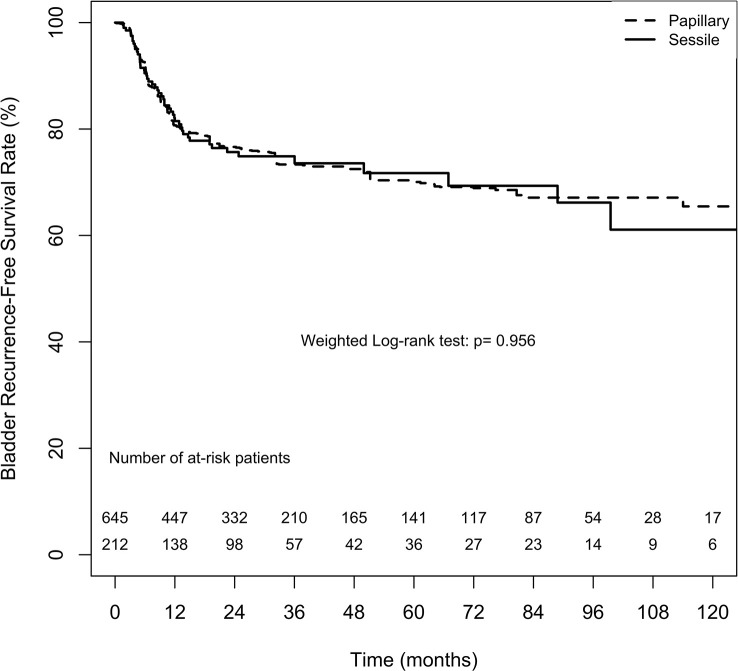
Average treatment effect for the treated (ATT) weighting Kaplan-Meier estimates for bladder recurrence-free survival rate. Numbers along x axis are the numbers of patients remaining in the risk set at each time point.

Bladder and contralateral recurrences were considered separately for analysing the recurrence-free survival rate. The overall 2-, 5-, and 10-year bladder recurrence-free survival estimates in the papillary tumor architecture group were 76.6% ( ± 3.1%), 70.0% ( ± 3.9%), and 65.5% ( ± 5.4%), respectively. Similarly, the overall 2-, 5-, and 10-year estimates for bladder recurrence-free survival in the sessile tumor architecture group were 75.7% ( ± 3.2%), 71.7% ( ± 3.9%), and 61.1% ( ± 6.9%), respectively. No significant difference in bladder recurrence-free survival rate between the groups was found either after weighting (weighted log-rank test, p = 0.956, **Figure 1**) or before weighting (unweighted log-rank test, p = 0.353, **Supplement 1**).

#### Contralateral Recurrence

**Figure 2:**
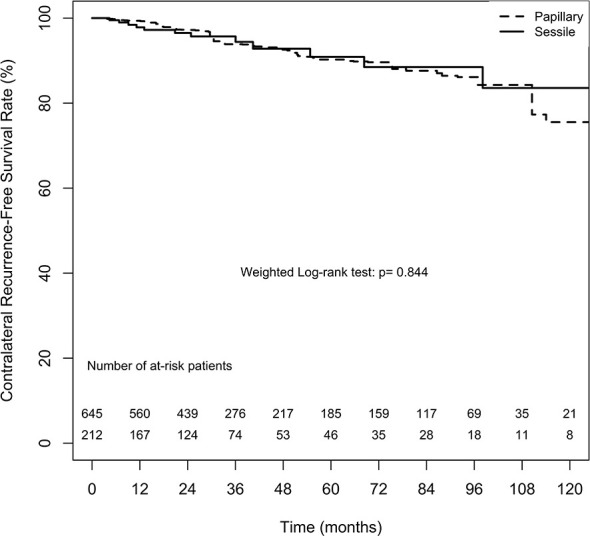
Average treatment effect for the treated (ATT) weighting Kaplan-Meier estimates for contralateral recurrence-free survival rate. Numbers along x axis are the numbers of patients remaining in the risk set at each time point.

The overall 2-, 5-, and 10-year contralateral recurrence-free survival estimates in the papillary tumor architecture group were 97.2% ( ± 1.3%), 90.2% ( ± 3.0%), and 75.5% ( ± 8.8%), respectively. By contrast, the overall 2-, 5-, and 10-year contralateral recurrence-free survival estimates in the sessile tumor architecture group were 96.5% ( ± 1.4%), 90.9% ( ± 3.2%), and 83.6% ( ± 6.0%), respectively. No significant difference in contralateral recurrence-free survival rate between the groups was found (weighted log-rank test, p = 0.844, **Figure 2**). The difference between the groups before weighting was also nonsignificant (unweighted log-rank test, p = 0.453, **Supplement 2**).

#### Metastasis

**Figure 3:**
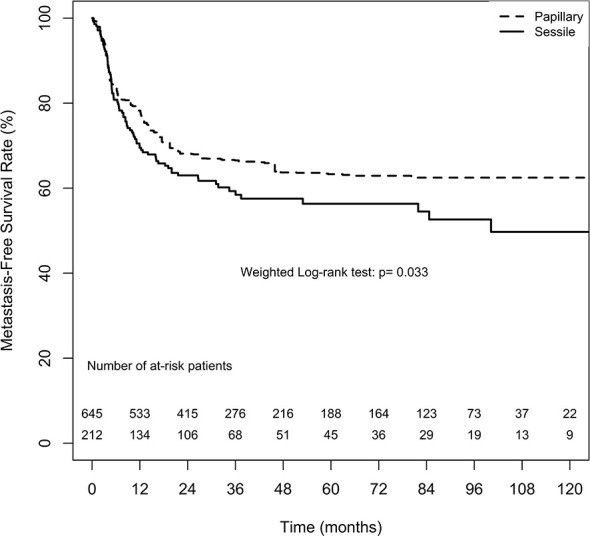
Average treatment effect for the treated (ATT) weighting Kaplan-Meier estimates for metastasis-free survival rate. Numbers along x axis are the numbers of patients remaining in the risk set at each time point.

The overall 2-, 5-, and 10-year MFS estimates in the papillary tumor architecture group were 68.1% ( ± 3.3%), 63.3% ( ± 3.7%), and 62.5% ( ± 3.8%), respectively. Nevertheless, the overall estimates in the sessile tumor architecture group at 2, 5, and 10 year were 63.0% ( ± 3.5%), 56.3% ( ± 3.9%), and 49.7% ( ± 5.0%), respectively. **Figure 3** demonstrates ATT weighted Kaplan–Meier estimates of MFS stratified by tumor architecture. The MFS rate was significantly lower in the sessile tumor architecture group both after (weighted log-rank test, p = 0.033) and before weighting (unweighted log-rank test, p < 0.001, **Supplement 3**).

#### Cancer-Specific Survival

**Figure 4:**
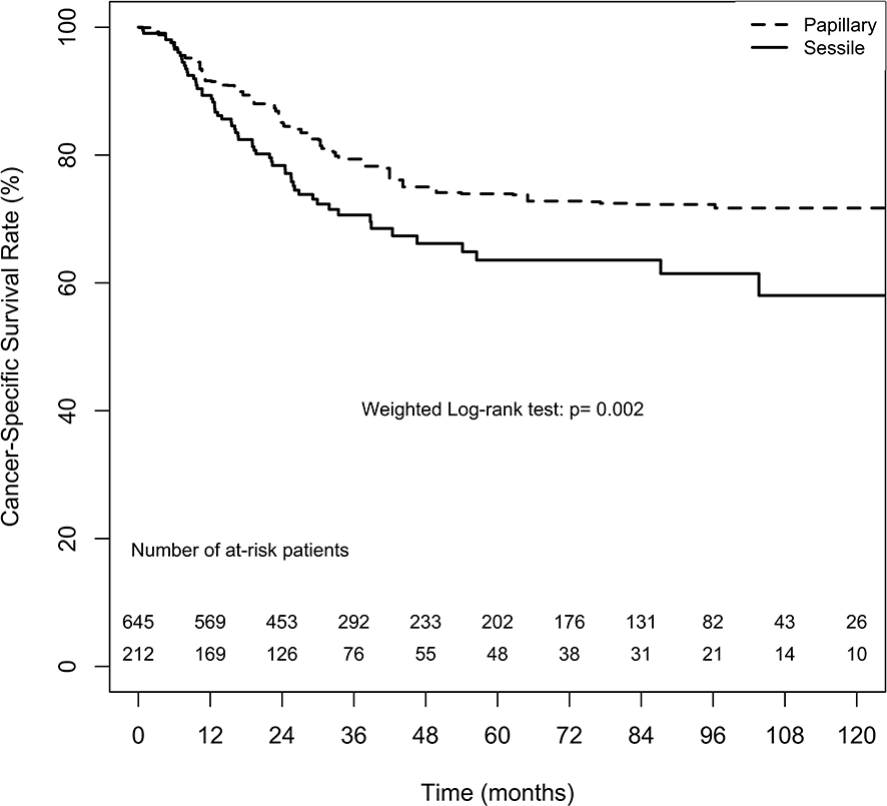
Average treatment effect for the treated (ATT) weighting Kaplan-Meier estimates for cancer-specific survival. Numbers along x axis are the numbers of patients remaining in the risk set at each time point.

The overall CSS estimates at 2, 5, and 10 years in the papillary tumor architecture group were 85.1% ( ± 2.6%), 73.9% ( ± 3.7%), and 71.7% ( ± 4.1%), respectively. Nevertheless, the overall estimates at 2, 5, and 10 years in the sessile tumor architecture group were 78.4% ( ± 3.0%), 63.6% ( ± 4.2%), and 58.0% ( ± 5.4%), respectively. **Figure 4** demonstrates ATT weighted Kaplan–Meier estimates of CSS stratified by tumor architecture. The survival rate was significantly lower in the sessile tumor architecture group (weighted log-rank test, p = 0.002). Additionally, the CSS rate was significantly lower in the sessile tumor architecture group before weighting (unweighted log-rank test, p < 0.001, **Supplement 4**).

## Discussion

First, this retrospective study supports the role of radical surgery for patients with localized UTUC. In our study, the 5-year bladder recurrence-free survival rate was 70% and 71.7% in papillary and sessile tumor architecture groups, respectively. The 5-year CSS rates in the papillary and sessile groups were 73.9% and 63.6%, respectively. Oncologic outcomes reported in our study were similar to those reported in other studies. In these studies, the recurrence rates in the bladder varied from 15% to 50% (20), and the 5-year CSS after RNU conducted in a 3-single-centre series ranged between 61% and 76% (2). Nevertheless, some patients experienced metastasis and cancer-related deaths after RNU. Therefore, we attempted to identify predictive factors for adverse outcomes and further develop the optimal therapeutic options.

The present study demonstrated that factors associated with the probability of tumor recurrence and death among patients with UTUC include pathological stage, histological grade, tumor architecture, lymphovascular invasion (LVI), and lymph node status (2, 3, 8, 9, 21). Additionally, neoadjuvant or adjuvant chemotherapy may influence the survival rate in this group of patients (2, 14). Consistent with previous studies, our data indicate the association of sessile tumor architecture with established features of biologically aggressive UTUC, such as advanced stage, high tumor grade, metastases to lymph nodes, and LVI (3, 5, 9). We also found a correlation of tumor architecture with bladder cancer history, CKD group, concomitant CIS, and the variant type. Notably, sessile tumor architecture was found to be associated with poor prognosis (3, 9, 22). We used propensity score weighting by ATT method to minimize the effect of confounding variables between two tumor architecture groups to precisely interpret the outcomes.

Approximately 24.7% of patients in our series exhibited sessile tumor growth pattern, whereas sessile architecture have been reported in 20%–28% of patients treated with RNU in other studies (2, 5). The rate of recurrence in the bladder after primary UTUC treatment has been reported to be 15%–50% (20). However, results indicating the association of tumor growth pattern with tumor recurrence are controversial. Some studies have concluded that tumor architecture is independently associated with disease recurrence (2–4, 23). Fan et al. reported a significantly lower recurrence-free survival (RFS) in patients with sessile architecture compared with those with papillary architecture, and the univariate and multivariate analyses have indicated that tumor architecture is an independent prognostic factor for RFS (6). Conversely, Fajkovic et al. (24) and Favaretto et al. (25) have not been able to establish the tumor architecture as a significant predictor for disease recurrence.

Several confounding factors, including patient-specific, tumor-specific, treatment-specific, and prognostic biomarkers have been shown to contribute to disease recurrence after RNU (6). The nonspecific relationships between tumor architecture and disease recurrence might account for negative findings in our study. In accordance with previous studies, we found that tumor architecture is significantly associated with the development of metastatic disease and an independent prognostic marker of CSS after RNU (2–4, 22). Indeed, tumor cell infiltration is a crucial step in tumor dissemination that facilitates further metastasis to distant organs (1). Similar to these studies, we found a strong association of tumor architecture with metastasis and CSS, indicating that sessile UTUC is more aggressive than papillary UTUC. Early diagnosis of these patients would allow a selective administration of adjuvant chemotherapy.

Studies have reported death of nearly 30% of patients with UTUC from metastasis within 5 years of RNU administration (2, 5). Early identification of patients at high risk of disease progression could therefore help tailor the follow-up protocols after surgery. In addition to well-known prognostic factors, such as stage, lymph node status, and grade, the tumor architecture may be a useful predictor for RNU outcomes. Moreover, the greatest advantage of this feature is that it can be accessed macroscopically during endoscopic examination. In a large multicentre series of more than 1,300 UTUC patients treated with RNU, Remzi et al. showed that macroscopic sessile architecture was independently associated with oncologic outcome. In a recent systemic review, sessile tumor architecture was considered to be a valuable biomarker for predicting prognoses of UTUC patients (3, 5, 26). Adequate risk-stratification is necessary for treatment selection, planning the follow-up, and enrolling patients into clinical trials for adjuvant therapy.

Our study has several limitations. First, because this was a retrospective, single-centre analysis, it has inherent limitations. Second, patients who had not received surgery were excluded. Third, the treatment of each patient by different physicians might have introduced differences despite the evaluation of specimens by pathologists specialized in urology; however, our findings are applicable because differences in practice patterns among the physicians in our study were reflective to those used in the real world. Finally, this study lacked the record of the number of lymph nodes removed and the operative method used. Furthermore, not all of the patients received lymph node dissection during surgery.

In conclusion, the tumor architecture of UTUC after RNU is associated with established features of aggressive disease and predictors of metastasis and CSS; however, it is not an independent risk factor for bladder or contralateral disease recurrence. For better appraisal of the course of UTUC, tumor architecture should be considered in a predictive model for disease progression and as a useful factor to identify patients who might benefit from close follow-up or early administration of systemic therapy. To reach any definitive conclusion regarding the prognostic value of tumor architecture, further confirmation using adequately designed prospective trials with larger sample sizes is required.

## Data Availability Statement

The raw data supporting the conclusions of this article will be made available by the authors, without undue reservation.

## Author Contributions

H-YL: acquisition of data, analysis and interpretation of data, drafting the manuscript, and statistical analysis. YTC: administrative, technical, and material support. S-CH: administrative, technical, and material support. H-JW: administrative, technical, and material support. YTC: administrative, technical, and material support. CK: administrative, technical, and material support. WL: administrative, technical, and material support. YS: administrative, technical, and material support. CH: administrative, technical, and material support. YLC: administrative, technical, and material support. YCC: administrative, technical, and material support. HL: conception and design and supervision. PC: conception and design and supervision. All authors contributed to the article and approved the submitted version.

## Funding

This study is funded by the project of Kaohsiung Chang Gung Memorial Hospital (CMRPG8J0921).

## Conflict of Interest

The authors declare that the research was conducted in the absence of any commercial or financial relationships that could be construed as a potential conflict of interest.
