# Supplementary material for: The Prognostic Impact of Tumor Architecture for Upper Urinary Tract Urothelial Carcinoma: A Propensity Score-Weighted Analysis
Source: Front Oncol. 2021 Feb 25;11:613696. doi: 10.3389/fonc.2021.613696 (PMC7947797; doi:10.3389/fonc.2021.613696)
Supplement: Supplementary file 1 [file DataSheet_1.docx]

Supplementary Material

Supplement 1. Kaplan-Meier estimates for bladder recurrence-free survival. Numbers along x axis are the numbers of patients remaining in the risk set at each time point.


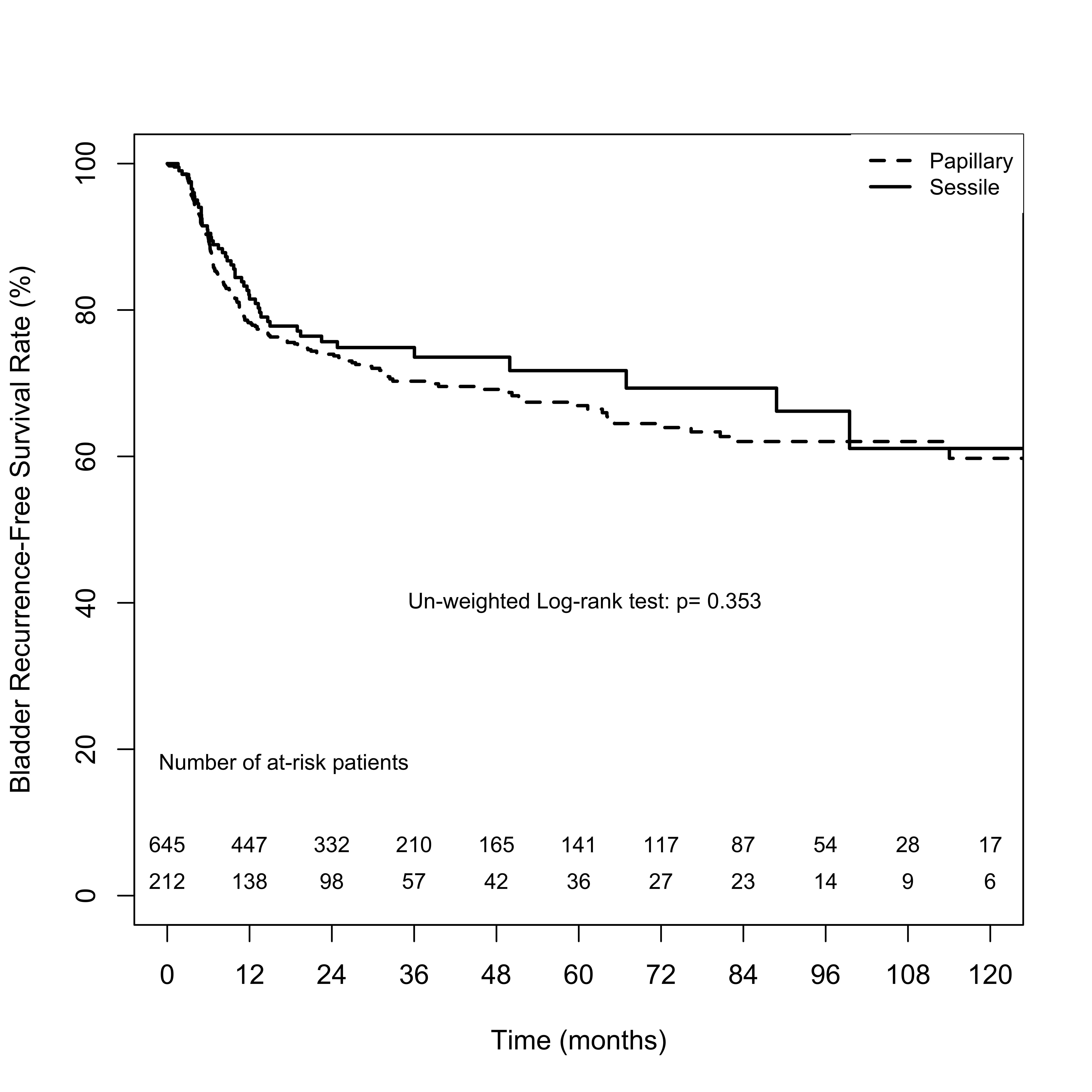
Supplement 2. Kaplan-Meier estimates for contralateral recurrence-free survival. Numbers along x axis are the numbers of patients remaining in the risk set at each time point.


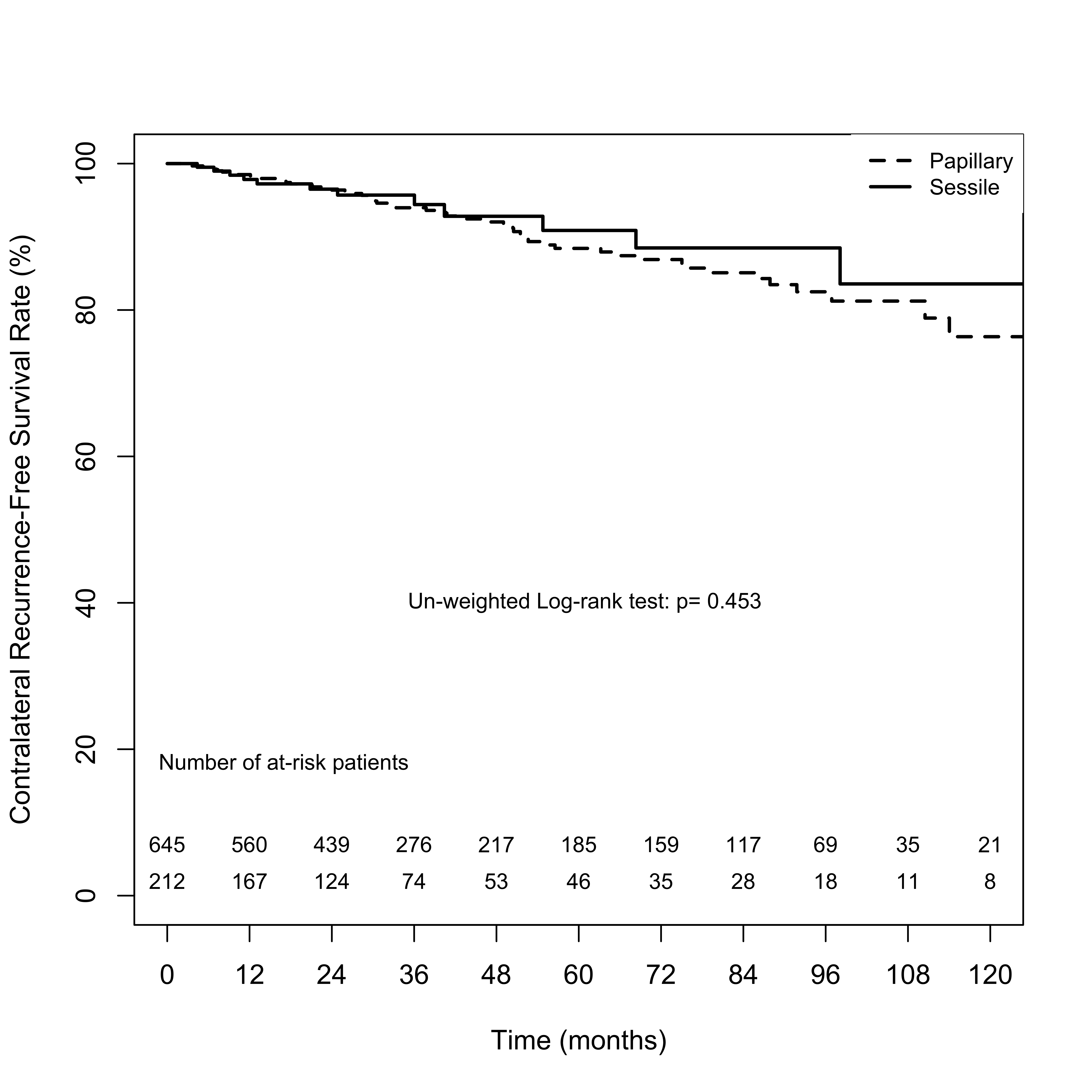


Supplement 3. Kaplan-Meier estimates for metastasis-free survival. Numbers along x axis are the numbers of patients remaining in the risk set at each time point.


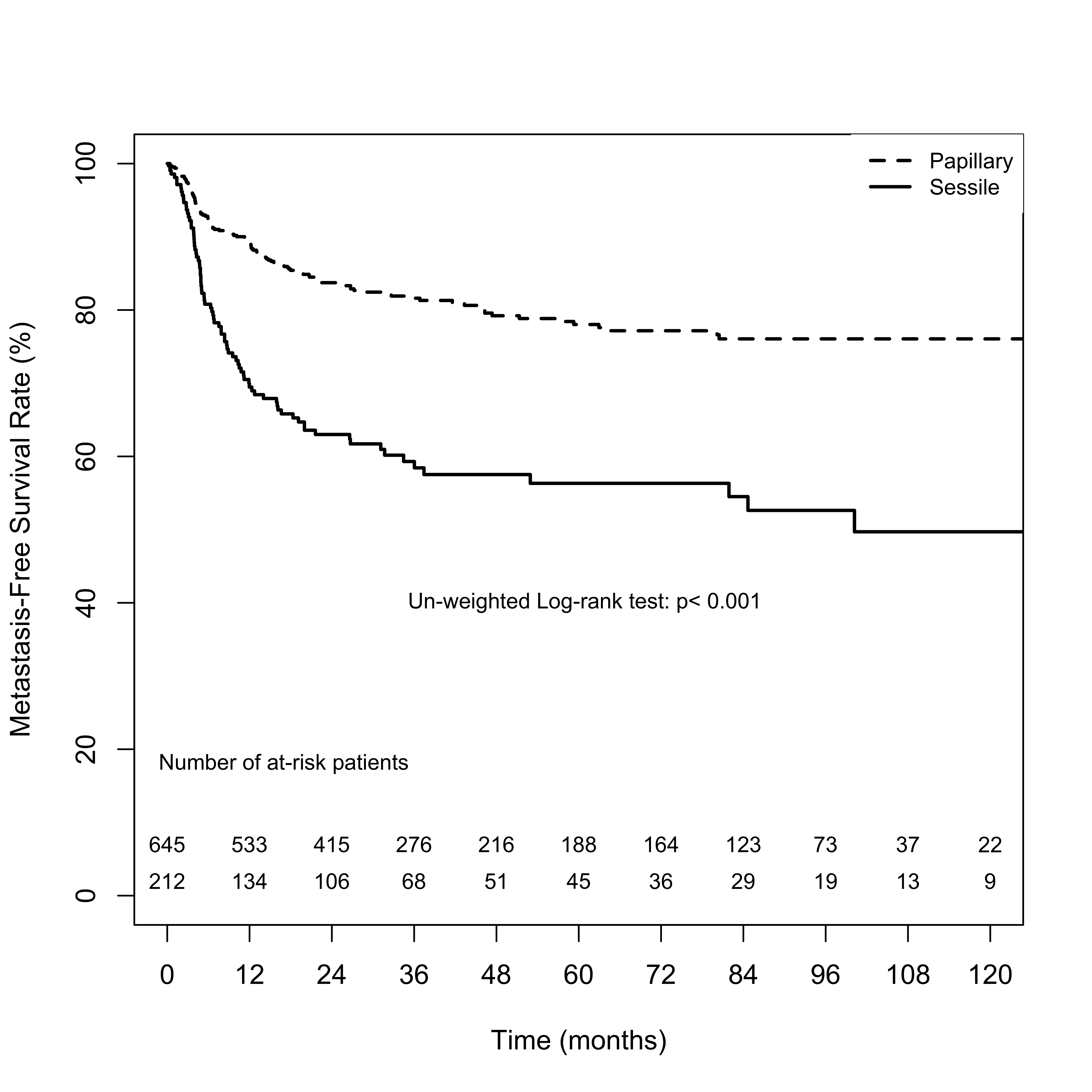


Supplement 4. Kaplan-Meier estimates for cancer-specific survival. Numbers along x axis are the numbers of patients remaining in the risk set at each time point.


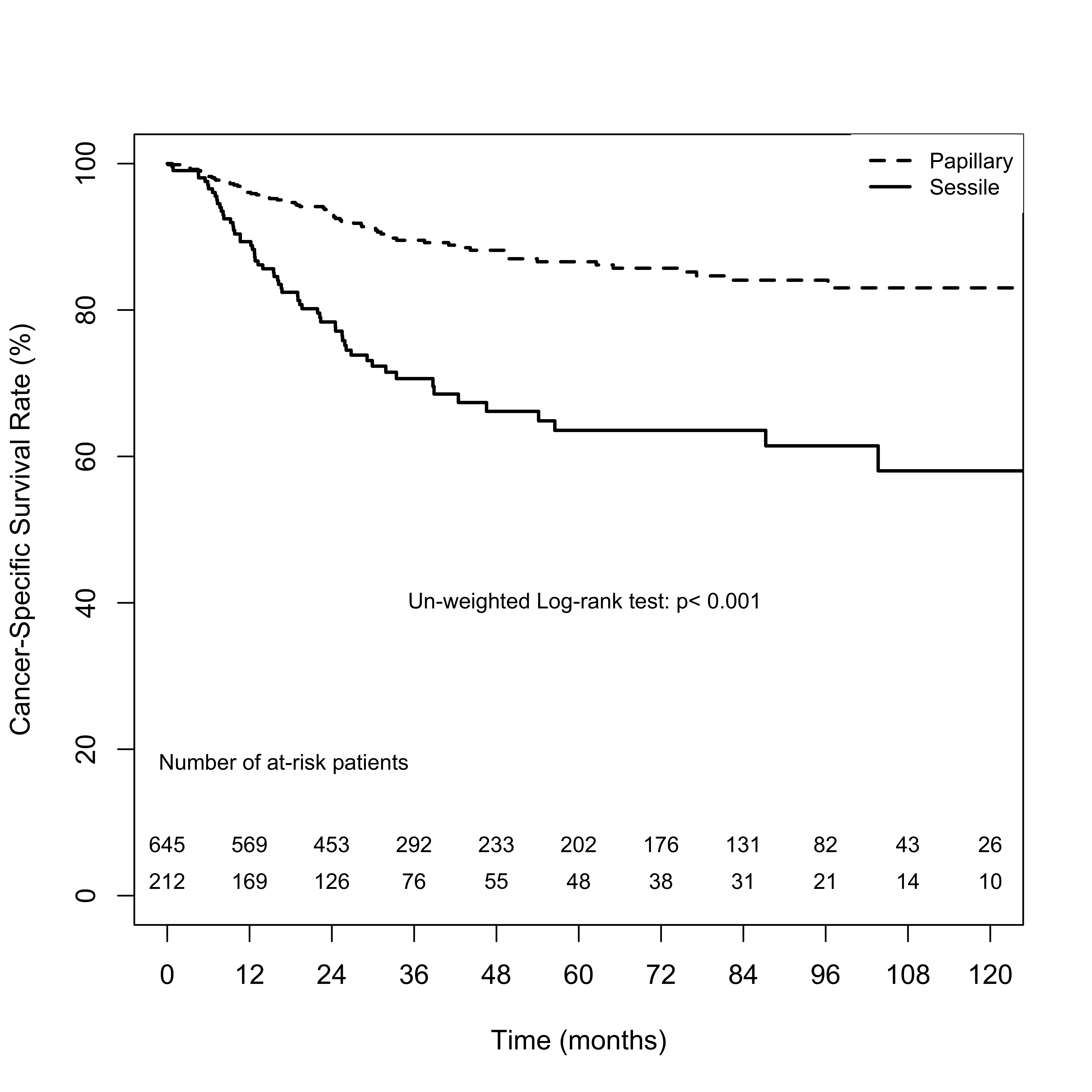


Supplement 5. Cumulative Bladder Recurrence-Free Survival Rate After Radical Nephroureterectomy

| Follow-up (Month) | Papillary tumor architecture | Sessile tumor architecture |
| --- | --- | --- |
|  | Probabilities ± SD (%) | Probabilities ± SD (%) |
| 12 | 80.7 ± 2.8 | 81.5 ± 2.8 |
| 24 | 76.6 ± 3.1 | 75.7 ± 3.2 |
| 36 | 73.3 ± 3.4 | 74.9 ± 3.3 |
| 48 | 72.5 ± 3.5 | 73.6 ± 3.5 |
| 60 | 70.0 ± 3.9 | 71.7 ± 3.9 |
| 72 | 68.9 ± 4.0 | 69.3 ± 4.4 |
| 84 | 67.1 ± 4.4 | 69.3 ± 4.4 |
| 96 | 67.1 ± 4.4 | 66.2 ± 5.2 |
| 108 | 67.1 ± 4.4 | 61.1 ± 6.9 |
| 120 | 65.5 ± 5.4 | 61.1 ± 6.9 |

Supplement 6. Cumulative Contralateral Recurrence-Free Survival Rate After Radical Nephroureterectomy

| Follow-up (Month) | Papillary tumor architecture | Sessile tumor architecture |
| --- | --- | --- |
|  | Probabilities ± SD (%) | Probabilities ± SD (%) |
| 12 | 99.4 ± 0.6 | 97.8 ± 1.1 |
| 24 | 97.2 ± 1.3 | 96.5 ± 1.4 |
| 36 | 93.9 ± 2.1 | 95.7 ± 1.6 |
| 48 | 92.6 ± 2.4 | 92.8 ± 2.6 |
| 60 | 90.2 ± 3.0 | 90.9 ± 3.2 |
| 72 | 89.6 ± 3.2 | 88.5 ± 3.9 |
| 84 | 87.6 ± 3.6 | 88.5 ± 3.9 |
| 96 | 86.1 ± 4.1 | 88.5 ± 3.9 |
| 108 | 84.3 ± 5.0 | 83.6 ± 6.0 |
| 120 | 75.5 ± 8.8 | 83.6 ± 6.0 |

Supplement 7. Cumulative Metastasis-Free Survival Rate After Radical Nephroureterectomy

| Follow-up (Month) | Papillary tumor architecture | Sessile tumor architecture |
| --- | --- | --- |
|  | Probabilities ± SD (%) | Probabilities ± SD (%) |
| 12 | 78.2 ± 2.9 | 69.5 ± 3.3 |
| 24 | 68.1 ± 3.3 | 63.0 ± 3.5 |
| 36 | 66.5 ± 3.4 | 59.3 ± 3.6 |
| 48 | 63.7 ± 3.6 | 57.5 ± 3.7 |
| 60 | 63.3 ± 3.7 | 56.3 ± 3.9 |
| 72 | 62.9 ± 3.7 | 56.3 ± 3.9 |
| 84 | 62.5 ± 3.8 | 54.5 ± 4.1 |
| 96 | 62.5 ± 3.8 | 52.6 ± 4.4 |
| 108 | 62.5 ± 3.8 | 49.7 ± 5.0 |
| 120 | 62.5 ± 3.8 | 49.7 ± 5.0 |

Supplement 8. Cumulative Cancer-Specific Survival Rate After Radical Nephroureterectomy

| Follow-up (Month) | Papillary tumor architecture | Sessile tumor architecture |
| --- | --- | --- |
|  | Probabilities ± SD (%) | Probabilities ± SD (%) |
| 12 | 91.6 ± 2.0 | 89.3 ± 2.2 |
| 24 | 85.1 ± 2.6 | 78.4 ± 3.0 |
| 36 | 79.4 ± 3.1 | 70.6 ± 3.5 |
| 48 | 75.0 ± 3.6 | 66.2 ± 3.9 |
| 60 | 73.9 ± 3.7 | 63.6 ± 4.2 |
| 72 | 72.8 ± 3.8 | 63.6 ± 4.2 |
| 84 | 72.3 ± 3.9 | 63.6 ± 4.2 |
| 96 | 72.3 ± 3.9 | 61.4 ± 4.6 |
| 108 | 71.7 ± 4.1 | 58.0 ± 5.4 |
| 120 | 71.7 ± 4.1 | 58.0 ± 5.4 |
